# Efficacy of deferred dosing of granulocyte colony-stimulating factor in autologous hematopoietic transplantation for multiple myeloma

**DOI:** 10.1038/bmt.2013.149

**Published:** 2013-10-07

**Authors:** J E Cox, S Campos, J Wu, R May, H Liu, C A Ramos, G Carrum, H E Heslop, M K Brenner, R T Kamble

**Affiliations:** 1 Department of Pharmacy, Center for Cell and Gene therapy, The Methodist Hospital, Houston, TX, USA; 2 Center for Cell and Gene therapy, Baylor College of Medicine, The Methodist Hospital, Houston, TX, USA

**Keywords:** multiple myeloma, autologous transplant, G-CSF

## Abstract

Routine administration of G-CSF following autologous hematopoietic SCT (ASCT) expedites ANC recovery and reduces hospitalization by 1–2 days; it has no impact on febrile neutropenia, infections, morbidity, mortality, event-free survival or OS. To determine whether delayed G-CSF dosage could result in equivalent ANC recovery and thereby improve cost effectiveness, we deferred the administration of G-CSF until WBC recovery had begun. A total of 117 patients with multiple myeloma received ASCT from January 2005 to September 2012. Of these, 52 were in the conventional dosing group (CGD) and received G-CSF from Day +7 for a median of five doses. In the deferred dosing group (DGD), 65 patients received G-CSF from median day 14 post transplant for a median of zero doses. There was no difference between groups in the incidence or duration of febrile neutropenia, duration of ⩾grade III mucositis, weight gain, rash, engraftment syndrome or early death (100 days). The DGD group had a significantly longer time to neutrophil engraftment than the CGD group (15 days vs 12 days; *P*<0.0001), a longer period of severe neutropenia (<100/μL; 8 days vs 6 days; *P*<0.0001), longer treatment with intravenous antibiotics (7 days vs 5 days; *P*=0.016) and longer hospital stay (19 days vs 17 days; *P*=<0.0001). Although the cost of G-CSF was lower in the DGD group (mean $308 vs $2467), the additional hospitalization raised the median total cost of ASCT in this group by 17%. There was, however, no adverse effect of deferred dosing on the rate of febrile neuropenic episodes or Day 100 survival, so that deferred dosing of G-CSF may be suitable for patients receiving ASCT as outpatients, for whom longer hospital stay would not be an offsetting cost.

## Introduction

Routine administration of G-CSF is a common practice following autologous hematopoietic SCT (ASCT). When administered from day +7 until ANC recovery >1000/μL, G-CSF expedites ANC recovery and reduces hospitalization by 1–2 days.^1, 2^ In most studies, however, administration has had no significant impact on febrile neutropenia, infections, morbidity, mortality, event-free survival or OS.^1, 2, 3, 4, 5, 6^ Whereas the American Society of Clinical Oncology (ASCO) recommends post-ASCT administration of G-CSF,^7^ the European Society for Medical Oncology (ESMO) describes it as controversial.^8^ Measures of costs and adverse effects are therefore imperative in assessing whether or not to continue to administer the agent routinely.

ASCT is an established treatment option for patients with multiple myeloma.^9, 10^ Standard of care prior to transplantation now uses agents with less myelosuppressive activity than oral melphalan, the agent most widely used in the past. Moreover, CD-34^+^ cell collection techniques have improved^11^ and alternative agents such as plerixafor are now available to assist mobilization where G-CSF/chemotherapy alone has failed.^12^ As a consequence, the median number of infused CD-34^+^ cells/kg has significantly increased over the past decade at our own center (3.79 (2.61–9.42) vs 4.49 (2.49–10.2, *P*=0.021).

Given this change, together with the improvements in post-transplant supportive care, it may no longer be appropriate to rely on earlier studies that showed reduced hospital stay for patients routinely receiving G-CSF to dictate the current standard of practice. We therefore stopped the routine use of G-CSF in January 2010 and adapted an alternative dosing schedule in which G-CSF was given only to accelerate neutrophil recovery once this had begun (>200/μL), and if subsequent increases to levels required for discharge (>500/μL) did not follow within 48 h. G-CSF optionally administered in this fashion was labeled deferred G-CSF dosing (DGD). We have now compared the outcomes of 65 patients who received autologous stem cell treatment for multiple myeloma in the DGD group with the outcomes in 52 patients with the disease who routinely received G-CSF daily from day 7 (conventional G-CSF dosing—CGD). We have compared each group for engraftment, complication rates, day of discharge and overall costs.

## Patients and methods

### Patient demographics

Following Institutional Review Board approval, we retrospectively analyzed data from 117 consecutive patients with multiple myeloma treated with ASCT between January 2005 to September 2012. Fifty-two patients were in the CGDgroup and 65 in the deferred G-CSF dosing (DGD) group. Patient, disease and transplant-related variables are described in Table 1. The baseline characteristics including age, sex, body weight, multiple myeloma stage, conditioning regimen and source of stem cells were well balanced between the two groups. More patients in the CGD group had more than two lines of therapy (58% vs 31% *P*=0.003). More patients in the DGD group received plerixafor (55% vs 12% *P*=<0.0001) and patients in the DGD group received a higher CD-34^+^ cell dose (median 4.49 × 10^6^ vs 3.79 × 10^6^; *P*=0.021).

### High-dose chemotherapy

In 83 patients, melphalan was administered intravenously at 200 mg/m^2^. Patients with co-morbid conditions including renal failure received a reduced dose of melphalan (140 mg/m^2^; *n*=34). All patients received supportive care following institutional standard operating procedures.

### Stem cell collection and storage

Patients received G-CSF (filgrastim) at 10 μg/kg/day, which was continued until the completion of stem cell collection. Patients with CD-34^+^ cell counts of less than 10 μL on the fourth day of mobilization received plerixafor (Mozobil, Genzyme Corp, Cambridge, MA, USA) at 24 mg/day, which was continued as necessary to completion of collection. Patients with creatinine clearance <60 mL/h received a reduced plerixafor dose (16 mg/day). Twenty-two of 117 (CGD *n*=20; DGD *n*=2) patients received chemotherapy-assisted mobilization using cyclophosphamide at 2–4 g/m^2^ followed by G-CSF (Table-1). PBSCs were collected using an Optia or Cobe Spectra system (Lakewood, CO, USA). Intermediate large-volume aphaeresis was performed at 12 L per session and PBSCs were cryopreserved at −196 °C in 10% DMSO using controlled rate freezing.

### Stem cell dose and post-transplant G-CSF dosing

The median dose of CD 34^+^ cells/kg was 3.79 × 10^6^/kg (2.61–9.42) in the CGD group and 4.49 × 10^6^/kg (2.49–10.2) in the DGD patients. Patients in the CGD group received daily doses of 5 μg/kg G-CSF beginning day +7 and those in the DGD group received 5 μg/kg doses at the physician's discretion following WBC recovery. DGD at physician's discretion is a optional alternative dosing schedule in which G-CSF is given only to accelerate WBC recovery once this had begun (>200/μL), and if subsequent increases to levels required for discharge (ANC >500/μL) did not follow within 48 hrs.

### Safety and efficacy end points

The primary end point was neutrophil engraftment (day post transplant on which ANC>500/μL × 3 consecutive days). Duration of severe neutropenia, time to platelets recovery to 20 000/μL and that to 50 000/μL, episodes of febrile neutropenia, regimen-related toxicity, duration of hospitalization and cost analysis were secondary end points (Table 2). Subgroup analysis was performed for patients receiving plerixafor for mobilization (*n*=42), those receiving >5.0 × 10^6^/kg CD 34^+^ cells (*n*=37) and those receiving no G-CSF in the DGD group (*n*=36).

Neutropenia was defined as an ANC of <500/μL, and temperature of 100.5 °F, defined as neutropenic fever. Severe neutropenia was defined as ANC of <100/μL. Platelet engraftment was defined as platelet counts of >20 000/μL for 3 consecutive days in the absence of platelet transfusions. Cost analysis included cost of G-CSF, cost of intravenous antibiotics and hospital charges for room and board (Table 2).

### Cost analysis

Our comprehensive cost analysis included an assessment of costs for the transplant admission, room and board, and drugs including antimicrobials and G-CSF. Total admission costs were calculated by combining costs from all cost centers including room and board, pharmacy, radiology, laboratory, central supply, blood bank and physician's fees. Costs for room and board were extracted from the admission cost for each patient. Drug costs reported for antimicrobials and G-CSF represent the average wholesale price (AWP) for each medication. Admission costs, including room and board and drug costs were compiled from The Methodist Hospital's centralized computer billing system. All reported costs represent actual costs for the administration of patient care as determined by the individual departmental finance sections.

### Statistical analysis

Descriptive statistics were calculated to describe baseline patient characteristics, transplant outcomes and cost assessment. Comparisons between the CGD and DGD groups were performed using the Wilcoxon rank-sum test for continuous variables and the χ^2^ or Fisher's exact test for categorical variables. Statistical significance was defined as *P*<0.05. All analyses were conducted using SAS 9.3.

## Results

### G-CSF timing and response

**Figure 1:**
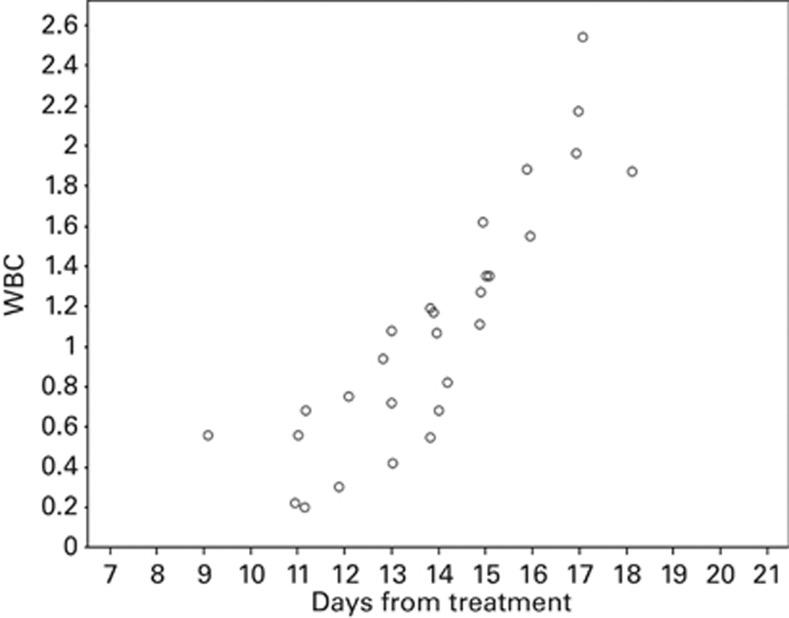
Timing of G-CSF administration in the DGD group (*n*=36).

Patients in the CGD group received more doses of G-CSF (median of 5 vs 0; *P*<0.0001) than patients in the DGD; indeed 36/65 (55%) patients in the DGD group received no G-CSF at all. The median post transplant day when G-CSF administration started in the DGD group was 14 days (range=9–18 days). The median total WBC counts and neutrophil counts at the first dose of G-CSF in the DGD group were 1080/μL (range=200–2540) and 148/μL (range=0–1168), respectively. The median ANC rise with the first dose of G-CSF in this group was 2203/μL (range=0–16 257/μL). Figure 1 illustrates the timing of G-CSF administration and WBC in the DGD group.

### Neutrophil and platelet recovery

As shown in Table 2, the DGD group had significantly prolonged time to neutrophil engraftment compared with the CGD group (15 days vs 12 days; *P*<0.0001) and a longer period of severe neutropenia (<100/μL), at 8 days vs 6 days (*P*<0.0001). There were no differences between groups in time to platelet engraftment or in their requirement for platelet transfusions.

### Effect of plerixafor assisted mobilization

**Figure 2:**
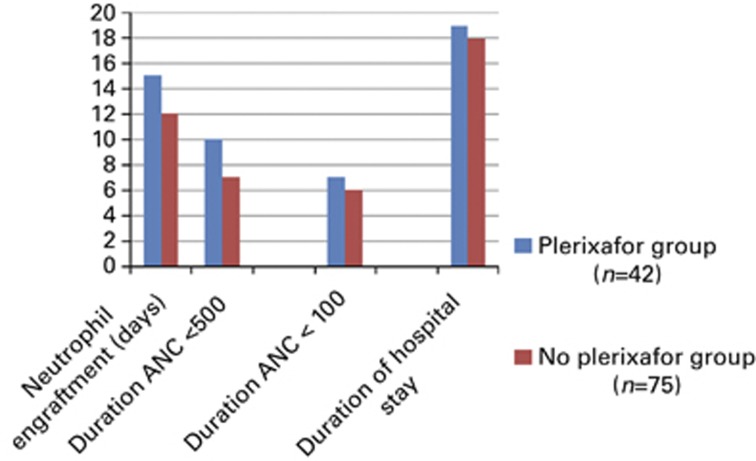
Outcomes on the *X* axis were inferior in plerixafor patients for number of days on the *Y* axis.

A total of 42 (DGD=36, CGD=6) patients received plerixafor to facilitate CD-34 mobilization. The outcomes of these patients were compared with those who did not receive plerixafor (*n*=75). As illustrated in Figure 2, there was a significant difference in the two groups all favoring patients who did not receive plerixafor. The neutrophil engraftment was faster (12 vs 15 days, *P*<0.0001), duration of severe neutropenia was improved (6 vs 7 days, *P*=0.008), duration neutropenia was significantly superior (7 vs 10 days, *P*<0.0001 and duration of hospital stay was shorter in patients who did not receive plerixafor (18 vs 19 days, *P*=0.034, Figure 2).

### Effect of CD-34 Cell dose

A total of 37 (31.6%) patients received >5.0 × 10^6^ CD-34 cells/kg. Mobilization strategies in these included G-CSF alone (n=14), G-CSF+chemotherapy (*n*=8) and G-CSF+plerixafor (n=15).The outcomes in these patients were compared with those receiving <5.0 × 106 CD-34 cells/kg (*n*=80). Interestingly, duration of neutropenia (9 vs 8 days, *P*=0.046), duration of severe neutropenia (7 vs 6 days, *P*=0.014) and duration of hospitalization (19 vs 18 days, *P*=0.028) were superior in patients receiving less than 5.0=106 CD-34 cells/kg. Of the 37 patients who received more than 5.0=106 CD34 cells/kg, 22 patients did not receive G-CSF+plerixafor mobilization. Compared to G-CSF+plerixafor mobilization, patients mobilized with G-CSF alone (*n*=14) or G-CSF+chemotherapy (*n*=8) had shorter duration of neutropenia (median 8 days), shorter duration of severe neutropenia (median 7 days) and shorter duration of hospitalization (median 16 days).

### Infection risk and antimicrobial utilization

Table 2 shows there were no significant differences between groups for the incidence of febrile neutropenia (60% vs 63%, *P*=0.702); the duration of febrile neutropenia (*P*=0.759); total number of antimicrobial drugs given (assessed as requirement for non-prophylactic antibacterial, antifungal or antiviral agents (*P*=0.597); or the incidence of cultures from any site positive for fungus or bacteria (*P*=0.338). However, the days of treatment with i.v. antibiotics was longer in the DGD group (7 days vs 5 days; *P*=0.016) (Figure 2).

### Toxicity and supportive care utilization

There were no deaths (by day 100) in either treatment group, and no significant difference in the incidence or duration of mucositis, weight gain, rash or bone pain.

### Duration of hospital stays and cost assessment

The duration of hospital stay was 2 days shorter in the CGD group (17 days vs 19 days; *P*=<0.0001). Despite the offsetting cost of additional G-CSF, the median total cost of ASCT for the CGD group was $61 547 (25 715–122 819) compared with $74 107 (33 104.27–112 213.44) for the DGD patients, a median difference of 17%.

## Discussion

We determined whether deferring G-CSF administration until the earliest signs of WBC recovery would reduce G-CSF utilization after autologous SCT (auto-SCT), without increasing morbidity or hospital stay, and thereby improve the cost-effectiveness of the approach. Our results indicate that routine administration of G-CSF given from day +7 onward to patients receiving auto-SCT for myeloma results in faster engraftment than a deferred dose schedule, and also shortens the duration of severe neutropenia. These effects result in significant cost savings because of a median 2-day reduction in length of hospital stay. Nonetheless, there was no difference between the two groups in the incidence of febrile neutropenia or its duration, in time to platelet recovery or the number of platelet transfusions, infections, mucositis, or in day 100 mortality. Our observation of greater antibiotic utilization in the DGD group even in the absence of any difference in rates of infection likely reflects the slower kinetics of neutophil recovery^13^ with resultant delay in reaching ANC of 500/μL to allow safe discontinuation of antibiotics.

Patients who received plerixafor-assisted mobilization had delayed engraftment, longer neutropenia and longer hospitalization compared with those who did not require plerixafor. As we offer plerixafor only to sub optimal mobilizers, the outcomes here likely select a population of patients who are 'hard to mobilize‘ and likely have accompanying slower engraftment kinetics.

Several earlier studies have compared the routine administration of G-CSF post transplant (that is, the CGD regimen) with placebo but a comparison of the efficacy and safety with a deferred dose regimen has not been made. Although our study of conventional vs deferred G-CSF administration is a retrospective analysis, the data are derived from a single center in which a relatively homogenous patient cohort has been treated following clinical standard operating procedures that were consistent over the study period (January 2005–September 2012), thereby minimizing the bias related to patient and treatment heterogeneity. Our results are similar to previous randomized placebo-controlled trials^1, 3, 4, 5, 13, 14^ and retrospective analyses^2, 6, 15, 16, 17, 18, 19^ in which G-CSF after ASCT accelerated engraftment and reduced both the administration of intravenous antibiotics and the duration of hospitalization. Most of these studies also demonstrated that early administration of G-CSF (filgrastim or its pegylated preparation) had no effect on the incidence of febrile neutropenia, infection rates, platelet recovery or transfusion, red cell recovery or transfusion, reduction in regimen-related toxicity or mortality. Our results are, however, different from Schmitz *et al.*,^4^ who reported reduced rate of infections in a randomized controlled trial of 192 patients (91% infection rate in the placebo group vs 67% in the G-CSF group). Unlike Bensinger *et al.*,^20^ we saw no evidence for a ‘platelet steal' phenomenon in recipients of G-CSF, as the recovery in both the CGD and DGD groups were identical.

Interestingly, receiving >5.0 × 106 CD34+ cells/kg did not facilitate engraftment. The duration of neutropenia, duration of severe neutropenia and duration of hospitalization was significantly superior in patients receiving less than 5.0 × 106 CD34+ cells/kg. The reason for this phenomenon is unclear. However, 15/37 (40%) patients have had mozobil mobilization, thus selecting a population of patients as discussed above. Although our patient size is small, these results are in contrast to a large trial studying CD34 cell dose-based engraftment kinetics.^13^

We recognize the retrospective nature of the study and its inherent shortcomings. Since we do not perform out patient transplants at our institution, we are not able to provide cost analysis for in patient vs out patient ASCT and its bearing on cost analysis for CGD or DGD

Although our results showed an overall cost benefit in recipients of CGD, the benefit was almost entirely attributable to the shorter period of hospitalization, which more than offset the savings from reduced G-CSF administration. As there was no adverse effect of deferred dosing on the rate of febrile neuropenic episodes or Day 100 survival, deferred dosing of G-CSF may be an attractive option for patients receiving ASCT on an outpatient basis in whom additional days of hospital stay would not be an offsetting factor.

## Figures and Tables

**Table 1:** Baseline characteristics

*Characteristics*	*CGD group*	*DGD group*	P-*value*
Number of patients	52	65	
Age	60 (35–75)	62 (37–79)	0.501
			
*Sex*			
Male	30 (58%)	36 (55%)	0.803
Female	22 (42%)	29 (45%)	
Weight, kg	83 (46–166)	78 (51–141)	0.165
			
*No. of prior chemotherapy courses*			
0–1	22 (42%)	45 (69%)	0.003
2+	30 (58%)	20 (31%)	
			
*Stem cell source*			
Peripheral blood	52 (100%)	65 (100%)	—
			
*Method of stem cell collection*			
G-CSF alone	26 (50%)	27 (42%)	<0.0001
G-CSF plus chemotherapy	20 (38%)	2 (3%)	
G-CSF plus Mozobil	6 (12%)	36 (55%)	
CD-34 dose ( × 10^6^)	3.79 (2.61–9.42)	4.49 (2.49–10.2)	0.021
			
*Conditioning regimen*			
Melphalan 200	39 (75%)	44 (68%)	0.387
Melphalan 140	13 (25%)	21 (32%)	

Abbreviations: CGD=conventional dosing group; DGD=deferred dosing group.

**Table 2:** Transplant outcomes in the CGD and DGD groups

*Characteristics*	*CGD group (median, range)*	*DGD group (median, range)*	P-*value*
Number of G-CSF doses	5 (1–8)	0 (0–5)	<0.0001
Neutrophil engraftment (days)	12 (11–14)	15 (11–20)	<0.0001
Duration of severe neutropenia (ANC<100) days	6 (4–9)	8 (4–10)	<0.0001
Duration of neutropenia (ANC<500)	7 (5–9)	10 (6–16)	<0.0001
Febrile neutropenia	33 (63%)	39 (60%)	0.702
Duration of fever	1 (1–9)	2 (1–5)	0.656
Duration of febrile neutropenia	1 (1–9)	1 (1–5)	0.759
Days to platelets 20 000 uL	17 (10–25)	17 (9–35)	0.472
Days to platelets 50 000 uL	18 (12–25)	17 (11–35)	0.476
Number of platelet units transfused	1 (0–6)	1 (0–7)	0.307
Duration of intravenous antibiotics (days)	5 (3–20)	7 (4–15)	0.016
Patients with positive blood culture	9 (17%)	16 (23%)	0.338
Grade III, IV mucositis	14 (27%)	18 (28%)	0.926
Weight gain (>5%)	1 (2%)	2 (3%)	1.0
Rash	1 (2%)	4 (6%)	0.38
Early (<100 days) death	0 (0%)	0 (0%)	
Duration of hospital stay	17 (14–24)	19 (16–28)	<0.0001
Cost of G-CSF	2467	308	<0.0001
Cost of intravenous antibiotics	214	316	0.160
Room and board	19 261	21 527	<0.0001
Total cost of transplant	61 547	74 107	0.094
Cost savings	$12 560 in the CGD group (16.95%)		

Abbreviations: CGD=conventional dosing group; DGD=deferred dosing group.
